# miR394 Acts as a Negative Regulator of *Arabidopsis* Resistance to *B. cinerea* Infection by Targeting *LCR*

**DOI:** 10.3389/fpls.2018.00903

**Published:** 2018-07-03

**Authors:** Xing Tian, Liping Song, Yi Wang, Weibo Jin, Fudan Tong, Fangli Wu

**Affiliations:** Institute of Bioengineering, College of Life Sciences, Zhejiang Sci-Tech University, Hangzhou, China

**Keywords:** miR394, *LCR*, overexpression, *Botrytis cinerea*, susceptibility, *Arabidopsis thaliana*, transgenic plant

## Abstract

Gray mold of tomato is caused by the pathogen *Botrytis cinerea*. MicroRNAs play a crucial role in the biotic and abiotic stress responses of plants and regulate their targets by gene silencing. miR394 is an ancient and conserved miRNA in plants, and it participates in the regulation of plant development and stress responses. In our previous study, miR394 was found to respond to *B. cinerea* infection in tomato, but the roles and regulatory mechanisms of miR394 in *B. cinerea*-infected tomato remain unclear. miR394 was down-regulated in tomato in response to *B. cinerea* infection, showing an expression pattern opposite to the previous finding that miR394 was up-regulated in tomato cv. *Jinpeng 1* infected by *B. cinerea*. We obtained transgenic *Arabidopsis* overexpressing miR394, which resulted in low expression levels of its target *LEAF CURLING RESPONSIVENESS* (*LCR*). Leaf lesion size and trypan blue staining showed that miR394 overexpression led to increased sensitivity of transgenic *Arabidopsis* to *B. cinerea* compared to wild type. We also detected changes in the expression levels of stress-related miRNAs, including miR159, miR156, miR168, and miR172. In the transgenic plants, it indicated potential cross talk between these miRNAs and miR394, except for miR159. miR394 also enhanced the expression of *ARGONAUTE 1* (*AGO1*), *DSRNA-BINDING PROTEIN 4* (*DRB4*) and the RNA-binding protein gene *DAWDLE* (*DDL*), which are involved in the pathways of miRNA biosynthesis and regulation, suggesting that miR394 overexpression has a feedback effect on these genes. Our data indicate that overexpression of miR394 in *Arabidopsis* increased the susceptibility of plants to *B. cinerea* by affecting the expression of its target gene *LCR* along with a number of key genes involved in plant miRNA metabolism (*AGO1*). Thus, miR394 is a negative regulator of *Arabidopsis* resistance to *B. cinerea* infection by targeting *LCR*.

## Introduction

Small RNA plays a significant role by regulating gene expression during pathogen infection (Padmanabhan and Dinesh-Kumar, 2009; Katiyaragarwal and Jin, 2010; Weiberg et al., 2014; Weiberg and Jin, 2015; Joshi et al., 2016). MicroRNAs (miRNAs) are one class of sRNAs. After their primary transcripts (pri-miRNAs) are processed by Dicer or *DCL*, they are incorporated into Argonaute (AGO) proteins to form RISCs that target genes with fully or partially complementary sequences. miRNAs can induce post-transcriptional gene silencing via mRNA degradation or translational inhibition (Filipowicz et al., 2005).

It is well established that the miR^∗^ is a by product of miRNA biogenesis and is generally degraded rapidly in cells. However, some miR^∗^ species exist stably in cells and could also regulate the expression of their targets. Depending on whether they come from the 5′ or 3′ sides of the miRNA precursor, these stable miR^∗^ species were later designated as miR-5p or -3p, respectively. Zhang et al. (2011) reported that *Arabidopsis* AGO2 regulates innate immunity through miRNA393^∗^-mediated gene silencing. Manavella et al. (2013) found that miR171a^∗^ can bind to the RISC complex and trigger silencing of *SU(VAR)3-9 HOMOLOG8*. Endo et al. (2013) showed that maturation of AGO7-RISC requires cleavage of the miR390^∗^ strand to regulate the auxin signaling pathway via production of TAS3 trans-acting siRNAs (tasiRNAs). The length of the miRNA^∗^ species can also affect miRNA-mediated cleavage of RNA that triggers the production of secondary siRNAs (Manavella et al., 2012).

Recent studies have identified plant miRNAs responsive to fungal infection in a number of plant species. Li et al. (2014) reported that miR160, miR164 and miR168 were induced whereas miR439 and miR396 were down-regulated in resistant rice cultivars but not in susceptible cultivars infected by *M. oryzae*. MiR169, miR172 and miR398 were induced in both resistant and susceptible cultivars, suggesting their role in basal responses (Li et al., 2014). Furthermore, overexpression of miR160 in a susceptible rice cultivar led to enhanced disease resistance to *M. oryzae*, suggesting its involvement in the defense response against this particular fungal pathogen (Li et al., 2014).

miR394 is an ancient and conserved miRNA present in a large number of dicot and monocot plant species (Sunkar and Jagadeeswaran, 2008) including *Arabidopsis thaliana* (Song et al., 2012; Jian et al., 2013; Knauer et al., 2013; Litholdo et al., 2016; Armenta-Medina et al., 2017), garlic (Chand et al., 2016), *Hypericum perforatum L.* (Galla et al., 2013), agarwood (Gao et al., 2014), *Solanum lycopersicum* (Gu et al., 2010; Jin and Wu, 2015), *Saccharum* spp. (Helena et al., 2012), *Brassica napus* (Song et al., 2015; Joshi et al., 2016), *Zea mays Linn*. (Li et al., 2013), *Citrus sinensis* (Lu et al., 2014), *Glycine max (Linn.) Merr.* (Ni et al., 2012), cotton (Shweta and Khan, 2014), *Acacia mangium* (Siang and Ratnam, 2012), *Camellia sinensis* (Sun et al., 2017). miR394 targets the gene *LCR*, which encodes an F-box protein (SKP1-Cullin/CDC53-F-box) involved in the regulation of development in leaves, seeds and fruits (Song et al., 2012, 2015; Sun et al., 2017). Song et al. (2012) found that overexpression of miR394a/b results in a curled-up leaf phenotype, while overexpression of a miR394-resistant version of *LCR* may lead to a curled-down leaf phenotype. Sun et al. (2017) found that miR394 may regulate the synthesis of catechin in tea leaves. In *B. napus*, Song et al. (2015) reported the involvement of miR394 in the regulation of fruit and seed development, as miR394 overexpression alters the storage oil contents and composition. miR394 was also identified as a mobile signal in the shoot meristem protoderm, and it contributes to the stem cell competence of the distal meristem by inhibiting *LCR* (Knauer et al., 2013).

Our previous results showed that miR394 was up-regulated in response to *B. cinerea* infection in tomato (Jin and Wu, 2015), suggesting that miR394 might play an important role in the regulation of the *B. cinerea* response in tomato. However, further functional analysis is required to confirm the regulatory role of miR394 during the interaction of tomato with *B. cinerea*. In this study, we generated transgenic *Arabidopsis* plants overexpressing miR394 and used them to investigate the roles of miR394 in *B. cinerea* infection.

## Materials and Methods

### Plants, *B. cinerea* Inoculation

Tomatoes (*Solanum lycopersicum*) cv. *Micro Tom* seeds were seeded directly into the soil, 16 h light and 8 h dark, 23°C in greenhouse. *B. cinerea* was inoculated on fresh tomatoes, maintained at 19–22°C with humidity. Briefly, the surfaces of stems, leaves and fruits of two-month-old plants were inoculated with 10 ul of *B. cinerea* conidia solution (1% Tween-20 and 2 × 10^6^ spores ml^-1^) using a soft brush. Solution of 1% Tween-20 without *B. cinerea* was used for mock-infection. After inoculation, the plants were maintained at 85% relative humidity to ensure spore germination. *B. cinerea*-inoculated and mock-inoculated leaves were harvested at eight time points (0, 0.5, 1, 2, 6, 12, 24, and 72 h) after treatment with three biological replicates. In addition, four tissues (stems, leaves, flowers and fruits) were harvested from plants without *B. cinerea* inoculation. The samples were frozen in liquid nitrogen and stored at -80°C for transcript level analyses.

*Lcr* mutants are available from Arabidopsis Biological Resource Center (ABRC), including *lcr*-1 (SALK_016763),*lcr*-2 (SALK_136833c) and *lcr*-3 (SALK_118210). The seeds of *A. thaliana* were sterilized with 10% NaClO and suspended on 1/2 MS medium add appropriate antibiotic. After 8–10 days, transfer the green seedlings to soil. Three-week-old *Arabidopsis* was treated with the same condition as above. *B. cinerea*-inoculated and mock-inoculated leaves were harvested at 24 h with three biological replicates.

### DNA and RNA Extraction and Reverse Transcription

Genomic DNA was extracted using Plant Genomic DNA Extraction Kit (TIANGEN, Beijing, China). Total RNAs were extracted using TRNzol-A+ reagent (TIANGEN, Beijing, China) and dissolved in diethyl pyrocarbonate (DEPC)-treated water. Their concentrations were quantified using a Nano Drop 2000 spectrophotometer.

For total RNAs, reverse transcription was performed as follows. Equal quantities of total RNA (1 μg) were reverse-transcribed using the PrimeScript RT Reagent Kit with gDNA Eraser (Takara, Dalian, China) according to the manufacturer’s recommendations. A similar reaction without reverse transcriptase was performed as a control to confirm the absence of genomic DNA in subsequent steps.

For miRNAs, we added poly(A) using *E. coli* poly(A) polymerase (NEB, Beijing, China). 3′ RT-Primer (Invitrogen) was used as the reverse transcription primer for the following reverse transcription according to the manufacturer’s protocol.

### Quantitative PCR (qPCR)

SYBR Green PCR was performed according to the manufacturer’s instructions (NEB, Beijing, China). Briefly, 1 μl cDNA template was added to 5 μl Taq 2 × Master Mix (NEB, Beijing, China), 10 μM specific primers and ddH_2_O to a final volume of 10 μl. The reactions were pre-denatured for 3 min at 94°C, followed by 40 cycles of 94°C for 30 s and 58°C for 30 s. All reactions were performed in triplicate, and controls (no template) were included for each gene. The threshold cycle (CT) values were automatically determined by the ABI 7500 Real-Time PCR System (United States). Confirmation of amplicon specificity was based on the melt curve at the end of each run and by product visualization after electrophoresis on an 8% polyacrylamide gel. The fold-changes were calculated using the 2^-ΔΔ*C*_T_^ method, where ΔΔCT = (CT, target – CT, inner) Infection – (CT, target – CT, inner) Mock (Livak and Schmittgen, 2001). All of the oligos used in this study are listed in **Supplementary Table S1**.

### Gene Constructs and the Generation of Transgenic *Arabidopsis* Plants

A 191 bp fragment corresponding to pre-miR394 and including the fold-back structure was amplified from the cDNA of tomato leaves using specific primers that introduced *Bam*H *I* and *Xba* I restriction sites. The purified products were cloned into the pBIN438 expression vector downstream of the CaMV 35S promoter region to overexpress miR394 in *Arabidopsis*. The construct was introduced into *Agrobacterium tumefaciens GV3101* and transformed into *Arabidopsis* ‘*Columbia*’ (*Col-0*) ecotype plants using the floral dip method. The transgenic lines were selected with *kanamycin* and confirmed by PCR amplification. T2 progenies were used in subsequent assays.

### Trypan Blue Staining

Wild-type and transgenic *A. thaliana* leaves treated with *B. cinerea* were stained with a solution containing 0.0625% (m/V) trypan blue. After the decolorization treatment, the distribution of mycelium in the diseased tissue was observed with a microscope to detect the infection effect of *B. cinerea*.

### Statistical Analysis

The statistical analysis was performed with SPSS statistical software 22.0 (United States). All the results were expressed as the Means with SDs from three independent experiments. The *t*-test was selected and the *P*-values < 0.05 were considered statistically significant.

## Results

### Expression Patterns of miR394 and Its Primary Transcript in Different Tomato Tissues

miR394 is an ancient and conserved miRNA in plants. So far, sixty members of the miR394 family have been reported in 26 plant species (miRBase database version 21). Most of the miR394 family members are encoded by the 5′ side of the *MIR394* gene and share the same sequence (5′-UUGGCAUUCUGUCCACCUCC-3′). Three miR394 family members, mes-miR394c and vvi-miR394a/c, have two extra nucleotides (AU) at the 3′-end compared to the common miR394 sequence. The remaining 11 members are encoded by the 3′ side of the *MIR394* gene and come from seven plant species. They were named miR394-3p and have different sequences (**Supplementary Figure S1**).

**FIGURE 1:**
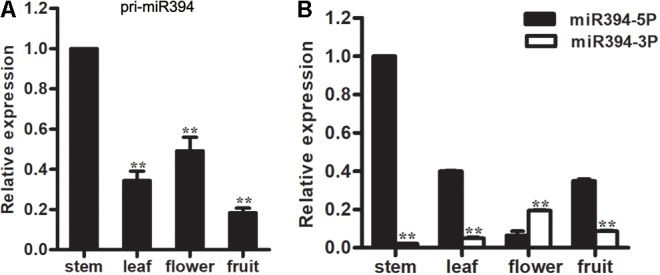
Expression analysis of miR394. **(A)** Expression levels of pri-miR394 in 4 different tissues. Asterisks indicate a significant difference (^∗∗^*P* < 0.01) compared with the stem. **(B)** Different expression levels in 4 tissues between miR394-5p and miR394-3p. Asterisks indicate a significant difference (^∗∗^*P* < 0.01).

To investigate the expression pattern of miR394, the abundance of the mature miRNA and its primary transcript was measured using qRT-PCR in the following four tomato tissues: stems, leaves, flowers and fruits. Both miR394 and its primary transcript (pri-miR394) were present in all four tissues, but the expression patterns varied. The level of pri-miR394 was lowest in fruits; highest in the stems; and intermediate in leaves and flowers (**Figure 1A**). For miR394-5p, the level was lowest in flowers; highest in stems; and intermediate in leaves and fruits (**Figure 1B**).

In addition, miR394-3p, originally designated as miR394^∗^, was also investigated in these four tomato tissues. The level of miR394-3p was lowest in stems; highest in flowers; and intermediate in leaves and fruits. Compared to miR394-5p, the expression levels of miR394-3p are noticeably lower in tomato tissues except flowers (**Figure 1B**). Therefore, miR394-5p was analyzed in the subsequent analyses.

### Expression Patterns of miR394 in Tomato Leaves Infected by *B. cinerea*

**FIGURE 2:**
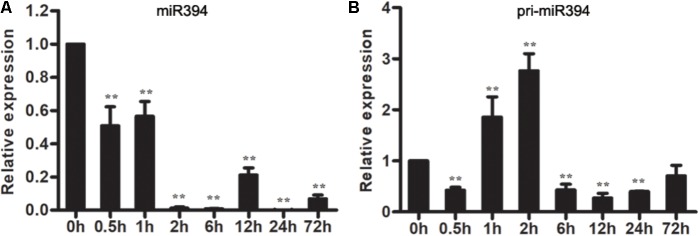
Expression patterns of miR394 **(A)** and pri-miR394 **(B)** in tomato leaves infected by *B. cinerea*. The asterisks indicate a significant difference (^∗^*P* < 0.05; ^∗∗^*P* < 0.01) compared with the corresponding controls 0 h.

To understand the response of miR394 in *B. cinerea*-infected tomato, the expression patterns of miR394 were measured in *B. cinerea*-infected tomato leaves. The abundance of miR394 has not significantly changed in the mock-infected tomato at different time points (**Supplementary Figure S3**), but decreased in *B. cinerea*-infected leaves (**Figure 2A**). Pri-miR394 also decreased in *B. cinerea*-infected leaves at all time points except for 1 and 2 hpi leaves, in which pri-miR394 was up-regulated (**Figure 2B**). These results confirmed that miR394 is a responsive factor in tomato infected by *B. cinerea*, with further analysis of miR394 function required to understand its regulatory role during the interaction of tomato with *B. cinerea*.

### Generation and Molecular Analysis of Transgenic *Arabidopsis* Overexpressing miR394

**FIGURE 3:**
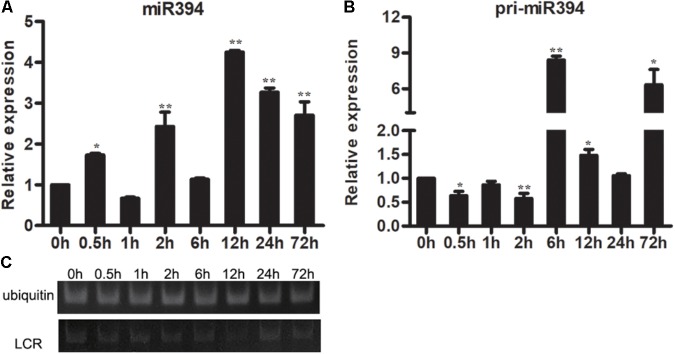
Expression patterns of miR394 **(A,B)** and *LCR* **(C)** in *Arabidopsis* leaves infected by *B. cinerea*. The asterisks indicate a significant difference (^∗^*P* < 0.05; ^∗∗^*P* < 0.01) compared with the 0 h. **(C)** The expression of *LCR* using semi-quantitative PCR.

The expression of miR394 was also investigated in *Arabidopsis* with or without *B. cinerea* infection. The result showed that the abundances of pri-miR394 were increased at 3 time points (6 h, 12 h and 72 h) in *Arabidopsis* after *B. cinerea* infection compared to 0 h (**Figure 3B**), whereas the abundances of miR394 was increased at 5 time points (0.5 h, 2 h, 12 h, 24 h and 72 h) (**Figure 3A**). Correspondingly, the expressions of *LCR* were decreased in *B. cinerea*-infected *Arabidopsis* (**Figure 3C**), showing a negative regulation with miR394.

**FIGURE 4:**
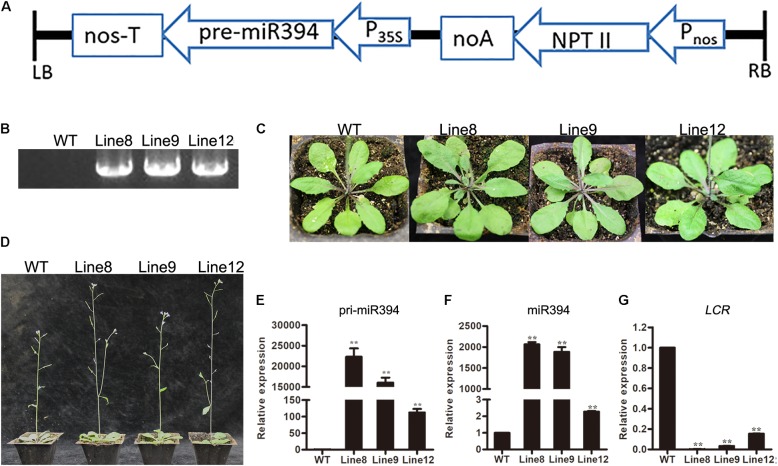
Analysis of transgenic *Arabidopsis* overexpressing miR394. **(A)** Overexpression construct diagram; **(B)** PCR analysis to amplify the *Kan* gene from genomic DNA; **(C)** leaf shape and **(D)** inflorescence in WT and different transgenic lines; **(E,F)** The expression level of primary transcript ath-miR394 and mature ath-miR394; **(G)** *LCR* mRNA transcript levels. Asterisks indicate a significant difference between the WT and the transgenic lines (^∗∗^*P* < 0.01).

To investigate the role of miR394 in tomato in response to *B. cinerea* infection, we generated a pre-miR394 overexpression construct and introduced it into wild-type (WT) *Arabidopsis* (*Col-0*) through *A. tumefaciens*-mediated transformation. *Arabidopsis* complementary DNA (cDNA) containing the miR394 precursor (pre-miR394) was amplified and cloned into the binary vector pBIN438, yielding the miR394 overexpression construct p35S-pre-miR394/pNOS-Kan. As diagrammed in **Figure 4A**, the *MIR394* gene was driven by the CaMV 35S promoter and linked to the *Kan* resistance gene driven by the nopaline synthase (NOS) promoter (pNOS). To confirm the selected plants as positive transgenic plants containing the miR394 overexpression construct, we amplified the *Kan* gene by PCR using genomic DNA (**Figure 4B**). To assess the roles of miR394, three *Kan*-positive T2 lines (Line 8, Line 9 and Line 12) were selected for subsequent analysis. The three transgenic lines were phenotypically similar to WT plants in terms of leaf shape and florescence (**Figures 4C,D**).

To verify the expression of ath-miR394 in the transgenic plants, RT-PCR was used to amplify primary ath-miR394 transcripts from cDNA, and the transcript levels were compared between the WT control and the three transgenic lines. The ath-miR394 primary transcripts were up-regulated in all of the selected transgenic lines compared to WT *Arabidopsis* (**Figure 4E**). To investigate whether pri-miR394 was properly processed into mature miRNA, we conducted qRT-PCR analysis and found that miR394 levels in the representative transgenic lines were significantly higher than in the WT control (**Figure 4F**). Thus, ath-miR394 was successfully expressed in the transgenic *Arabidopsis* lines. Generally, miRNAs regulate target genes by post-transcriptional regulation. In *Arabidopsis*, miR394 is known to target the transcription factor *LCR* (Jian et al., 2013; Song et al., 2016). We therefore quantified *LCR* transcript levels by qRT-PCR in miR394-overexpressing transgenic *Arabidopsis* and found decreased *LCR* mRNA transcript levels in the leaves of transgenic Line 8, Line 9 and Line 12 compared to the WT control (**Figure 4G**).

### Overexpression of miR394 Enhances Susceptibility to *B. cinerea* Infection

**FIGURE 5:**
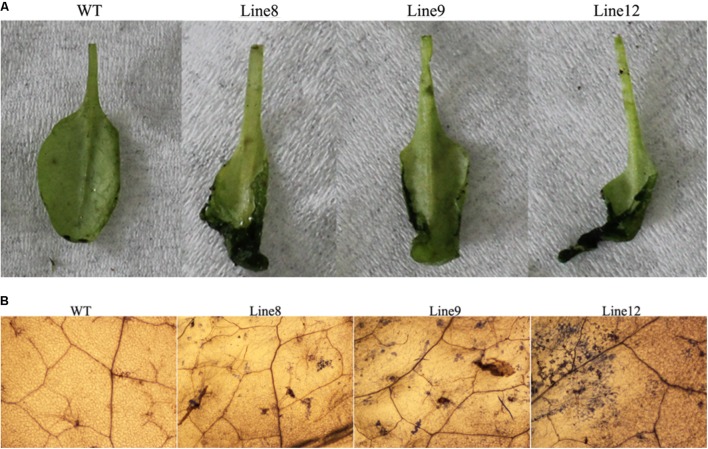
Enhanced susceptibility of transgenic *Arabidopsis* to *B. cinerea* infection. **(A)** Disease symptoms on the leaves after *B. cinerea* inoculation for 3 days; **(B)** trypan blue staining after *B. cinerea* inoculation for 3 days.

To determine whether ath-miR394 plays a role in the defense against pathogen infection, both WT and transgenic plants (Line 8, Line 9 and Line 12) were inoculated with *B. cinerea* for 3 days, and the physical appearance of the plants was assessed. The transgenic plants had larger necrotic spots than the WT plants (**Figure 5A**). Trypan blue staining further revealed that the *B. cinerea*-infected leaves of these three transgenic lines had more necrotic cells compared to WT (**Figure 5B**). These results suggest that overexpression of ath-miR394 enhanced the susceptibility of the transgenic plants to *B. cinerea* infection.

### Other miRNAs Are Affected in miR394-Overexpressing Transgenic Lines

**FIGURE 6:**
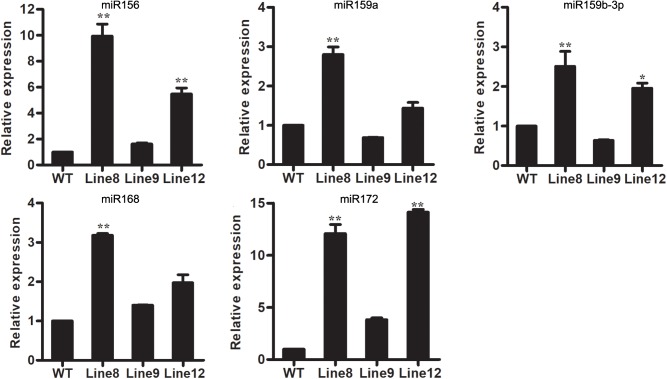
Expression levels of stress-related miRNAs in WT and transgenic lines. Asterisks indicate a significant difference between the WT and the transgenic lines (^∗^*P* < 0.05; ^∗∗^*P* < 0.01).

Although miRNAs have been found to play important roles in the complex stress response network, the molecular mechanisms underlying miRNA-mediated stress response are still unclear. In this study, we wanted to determine if different miRNAs interact in the plant response to *B. cinerea* stress. Several conserved miRNAs known to be involved in stress responses were selected for analysis, including miR159, miR156, miR168 and miR172 (Peng et al., 2010; Khraiwesh et al., 2012; Li et al., 2012, 2014; Gupta et al., 2014; He et al., 2014). MiR156, miR168 and miR172 were up-regulated in all transgenic plants overexpressing miR394 compared to WT, whereas miR159 was up-regulated in Line 8 and Line 12 but down-regulated in Line 9 (**Figure 6**). These findings suggest potential cross talk of miR394 with other miRNAs in the regulation of plant stress responses, except for miR159.

### miR394 Affects miRNAs Metabolism Pathway in miR394-Overexpressing Plants

**FIGURE 7:**
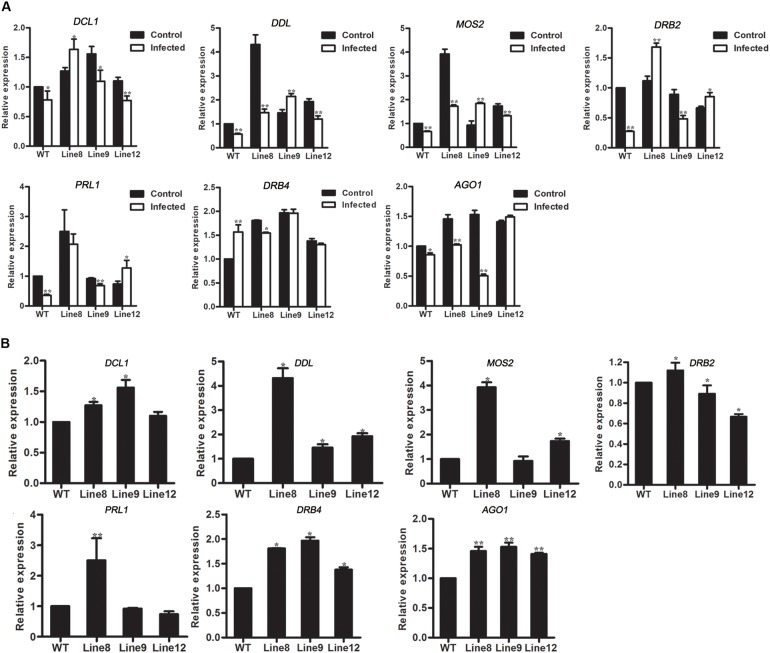
Expression levels of genes involved in miRNA metabolism. **(A)** Expression levels of *DCL1*, *DDL*, *MOS2*, *DRB2*, *PRL1*, *DRB4*, and *AGO1* in *Arabidopsis* without *B. cinerea* inoculated and with *B. cinerea* inoculated at 24 h, respectively. Asterisks indicate a significant difference between the control and the treatment (^∗^*P* < 0.05; ^∗∗^*P* < 0.01); **(B)** Expression levels of *DCL1*, *DDL*, *MOS2*, *DRB2*, *PRL1*, *DRB4* and *AGO1* in *Arabidopsis* with mock inoculation. Asterisks indicate a significant difference (^∗^*P* < 0.05; ^∗∗^*P* < 0.01) vs. WT.

To access the effects of genes in miRNA metabolism, the expressions of *AGO1* and 6 genes in miRNA biogenesis were investigated in transgenic plants. In mock-infected *Arabidopsis, AGO1* was up-regulated in miR394-overexpressing plants compared with WT (**Figure 7B**). However, *AGO1* transcripts was down-regulated in *B. cinerea*-infected plants (Line 8, Line 9 and WT but not Line 12) compared to the mock-infected plants (**Figure 7A**), consistent with the report of Weiberg et al. (2013), suggesting that miR394 enhances the susceptibility of transgenic plants to *B. cinerea* infection through its targeting of *LCR* and then affects an unknown pathway in *Arabidopsis*.

In addition to *AGO1*, the effect of miR394 overexpression on other miRNA biogenesis genes was also investigated. *DCL1* (*DICER-LIKE 1*), *DDL* (*DAWDLE*), *MOS2*, *DRB2*, *PRL1* and *DRB4* play important roles in miRNA biogenesis. The result showed that the expression patterns of these 6 genes were different between *Arabidopsis* with and without *B. cinerea* infection (**Figure 7A**). In addition, *DDL* and *DRB4* were up-regulated in the three transgenic lines without *B. cinerea* infection (**Figure 7B**).

### *Lcr* Mutant Enhances Susceptibility to *B. cinerea* Infection

**FIGURE 8:**
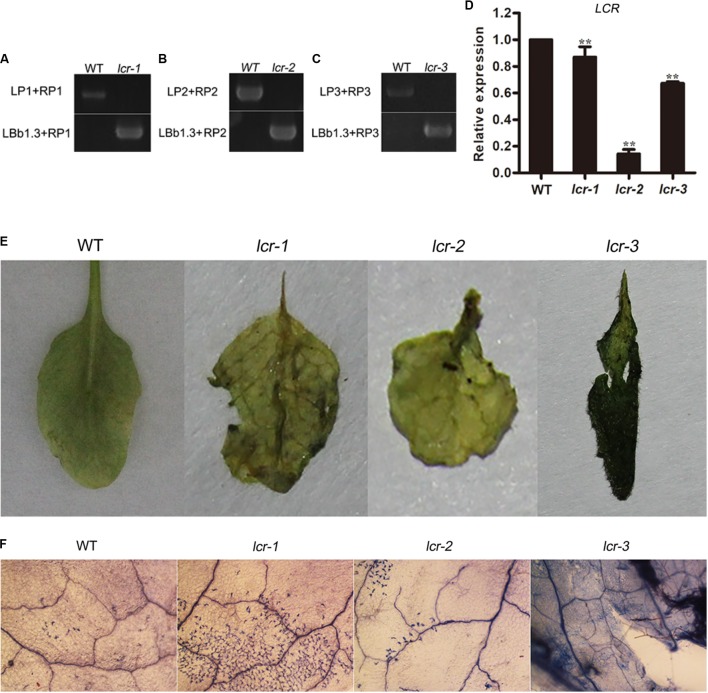
Enhanced susceptibility of *lcr* mutants to *B. cinerea* infection. **(A–C)** homozygous detection of *lcr* mutant. *LP+RP* for WT amplicons; *LBb1.3+RP* for mutant amplicons. **(D)** *LCR* expression level in mutants. Asterisks indicate a significant difference (^∗∗^*P* < 0.01) vs. WT. **(E)** Disease symptoms on the leaves after *B. cinerea* inoculation for 3 days; **(F)** Trypan blue staining after *B. cinerea* inoculation for 3 days.

To furtherly analyze the role of miR394 in *B. cinerea*-infected plant, three homozygous plants of *lcr* mutants were bought from ABRC and detected for the *B. cinerea* resistance. The result showed that *lcr* mutants had larger necrotic spots than the WT plants (**Figure 8**), showing a consistent phenotype with miR394 over-expressing plants. These results suggest that miR394 is a negative regulator of *Arabidopsis* resistance to *B. cinerea* infection by targeting *LCR*.

## Discussion

miR394-3p was listed as a miR^∗^ species in miRBase database version 18 and prior. Therefore, few reports have included the expression patterns of miR394-3p in different tissues and its functions in plants, even in *Arabidopsis*. In this study, the expression patterns of both miR394-5p and miR394-3p were investigated in different tissues in tomato (**Figure 1B**) and *Arabidopsis* (**Supplementary Figure S2**), and miR394-5p levels were much higher than miR394-3p levels in both species, except for flowers in tomato. While the expression patterns of both miR394-5p and miR394-3p were similar among different *Arabidopsis* tissues, they varied in tomato tissues.

*Leaf Curling Responsiveness*, which encodes an F-box protein (SKP1-Cullin/CDC53-F-box), is a unique target gene of miR394a and miR394b-5p; no target gene was found for miR394b-3p in *Arabidopsis*. Our previous study showed that tomato *Solyc05g015520.2.1*, which encodes an F-box protein, is a target gene of miR394 (Jin and Wu, 2015). Recently, miR394 and *LCR* were implicated in the regulation of leaf curling-related morphology in *Arabidopsis*. Specifically, overexpression of miR394-resistant *LCR* results in a curled-down leaf defect, whereas miR394a/b overexpression leads to a curled-up leaf phenotype (Song et al., 2012). In this study, the curled-up leaf phenotype was not observed in the miR394 overexpression lines compared to WT.

In addition to its effects on plant growth and development, miR394 is also induced by abiotic stresses in plants (Jonesrhoades and Bartel, 2004; Liu et al., 2008; Huang et al., 2010; Kong and Yang, 2010; Mendoza-Soto et al., 2012; Zhou et al., 2012). High levels of miR394 or low *LCR* activity may enhance tolerance to drought stress in transgenic plants (Helena et al., 2012; Ni et al., 2012; Jian et al., 2013). In contrast, high levels of miR394a/b and low *LCR* activities result in hypersensitivity to salt stress (Jian et al., 2013). When plants overexpressing miR394a and an *lcr* mutant were treated with cold, the results indicated that miR394 has a positive effect on cold stress, while *LCR* does not (Zhou et al., 2008; Song et al., 2016). Different time points after wounding of *Aquilaria sinensis* stem, miR394 expression decreased compared to the control (Gao et al., 2014). In *B. napus*, sulfate deficiency led to up-regulation of miR394 expression in roots and stems, and miR394 was found to be induced by cadmium in all tissues (Huang et al., 2010). In *A. thaliana*, miR394 is involved in iron deficiency (Kong and Yang, 2010). Dmitriev et al. (2017) found that miR394 expression increased after 4 h under aluminum stress but decreased after 24 h. Pi (phosphate) starvation caused miR394 to be up-regulated in tomato roots (Gu et al., 2010).

Recent studies have shown that miR394 plays critical roles in plant-microbe interactions. For example, the expression level of miR394 is lower in *AM* (*arbuscular mycorrhizal*)-treated tomato roots than the control (Gu et al., 2010). Under normal watering conditions, Italian white grape cv. Bosco (*Vitis vinifera L.*) treated with *Grapevine rupestris* stem pitting-associated virus (*GRSPaV*) had up-regulated miR394 expression levels compared to *GRSPaV*-free plants (Pantaleo et al., 2016). *Shen et al.* found that miR394 was induced in *B. napus* infected by the pathogenic fungus *Verticillium longisporum* (Dan et al., 2014). Chand et al. (2016) found that miR394 was up-regulated in response to *Fusarium oxysporum* f. sp. *cepae* (*FOC*) infection in garlic. In the same study, expression analysis revealed significant down-regulation of miR394 target genes in *FOC*-inoculated leaves, which indicated a negative relationship to the expression of miR394 (Chand et al., 2016). Our previous findings showed the up-regulation of miR394 in response to *B. cinerea* infection in tomato cv. *Jinpeng 1* (Jin and Wu, 2015), suggesting that miR394 may have an important role in regulating the response to *B. cinerea* in tomato. However, confirming the hypothesized regulatory role of miR394 during the interaction of tomato with *B. cinerea* required further functional analysis. In this study, we generated transgenic *Arabidopsis* plants overexpressing miR394 and used them to investigate the roles of miR394 in *B. cinerea* infection.

Emerging data has shown that the expression patterns of a gene might be different in 2 or more plant varieties with the same stress. Garg et al. (2015) showed that the expression patterns of many genes are different or even opposite in three rice varieties (IR64; Nagina 22; Pokkali) with drought stress. Du et al. (2017) reported that there are 314 transcriptional factor coding genes were differentially expressed in two maize varieties under salt stress. A putative disease-resistant protein (VIT_14s0030g00960) was down-regulated in “Shami” grapes and no significant change in “Beituni” grapes after drought (Saleh et al., 2017). The present findings show that miR394 was down-regulated in *B. cinerea*-infected tomato cv. *Micro Tom* (**Figure 2A**) but up-regulated in *B. cinerea*-infected *Arabidopsis* (**Figure 3A**); the findings in *Arabidopsis* were consistent with the previous findings in tomato cv. *Jinpeng 1* (Jin and Wu, 2015). The opposite miR394 expression patterns in different *B. cinerea*-infected plants made it difficult to confirm the resistance of this miRNA against *B. cinerea* in plants. In present study, we validated that miR394 is a negative regulator of defense by overexpressing miR394 in *Arabidopsis*.

**FIGURE 9:**
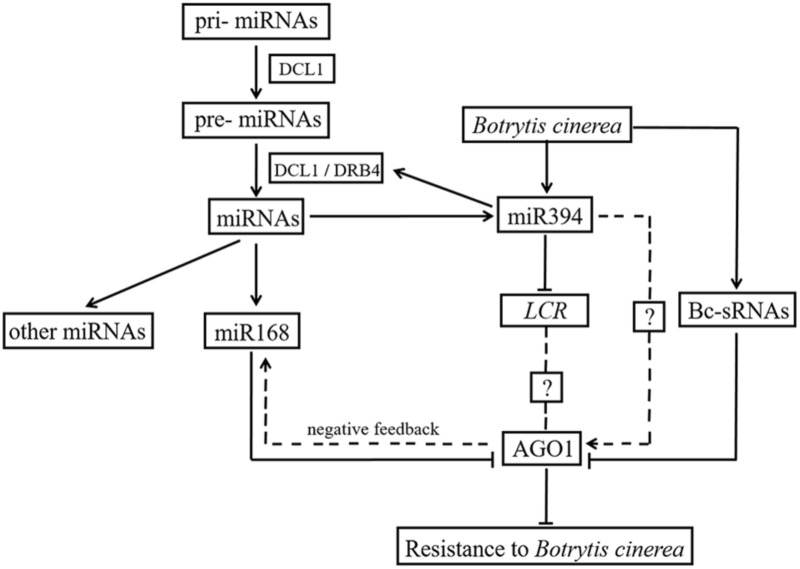
Cross talk between miR394 and other pathways.

Weiberg et al. (2013) previously reported that siRNAs encoded by *B. cinerea* can hijack plant immunity by suppressing the expression of host *AGO1*. In the present study, *AGO1* was up-regulated in two miR394 overexpression lines compared to WT under mock infection (**Figure 7B**), suggesting a positive effect of miR394 on *AGO1* expression. MiR168, which directly targets *AGO1* in plants (Li et al., 2012), was also up-regulated in the miR394 overexpression lines compared to WT (**Figure 6**), showing a positive correlation with its target gene *AGO1*. One possible explanation is that the high abundance of *AGO1* resulting from miR394 overexpression may have a positive feedback effect on miR168 expression in the transgenic plants. In addition to this finding, *AGO1* transcripts were not decreased in *B. cinerea*-infected transgenic plants compared with WT (**Figure 6**). Therefore, miR394 may enhance the susceptibility of transgenic plants to *B. cinerea* infection by targeting *LCR* and then affecting an unknown pathway in *Arabidopsis* (**Figure 9**).

## Author Contributions

WJ, XT, and LS designed and performed the experiments, analyzed the data and wrote the manuscript. FW contributed to treated plants with pathogen. YW constructed the over-expression vector. FT and WJ edited the manuscript. All authors read and approved the final manuscript.

## Conflict of Interest Statement

The authors declare that the research was conducted in the absence of any commercial or financial relationships that could be construed as a potential conflict of interest.

## Supplementary Material

FIGURE S1 Homolog of miR394 in plant species.

FIGURE S2 Different expression levels in 4 *Arabidopsis* tissues between miR394-5p and miR394-3p. Asterisks indicate a significant difference (^∗^*P* < 0.05).

FIGURE S3 Expression pattern of miR394 in mock-infected tomato leaves.

TABLE S1 Primers used.

## Abbreviations

*AGO1*: *ARGONAUTE 1*
*A. tumefaciens*: *Agrobacterium tumefaciens*
*B. cinerea*: *Botrytis cinerea*
*DCL*: *DICER-LIKE protein*
*DDL*: *RNA-binding protein DAWDLE*
*DRB2*: *DsRNA-Binding protein 2*
*DRB4*: *DsRNA-Binding protein 4*
*Kan*: *kanamycin*
*LCR*: *Leaf Curling Responsiveness*
miRNA: microRNA
miR**^∗^**: miRNA star strand
*MOS2*: *MODIFIER OF SNC1 2*
*M. oryzae*: *Magnaporthe oryzae*
*PRL1*: *PLEIOTROPIC REGULATORY LOCUS 1*
qRT-PCR: quantitative reverse transcription PCR
RISC: RNA-induced silencing complex
sRNAs: small RNAs
WT: wild type
