# Supplementary figures and images for: miR394 Acts as a Negative Regulator of Arabidopsis Resistance to B. cinerea Infection by Targeting LCR
Source: Front Plant Sci. 2018 Jul 3;9:903. doi: 10.3389/fpls.2018.00903 (PMC6037856; doi:10.3389/fpls.2018.00903)

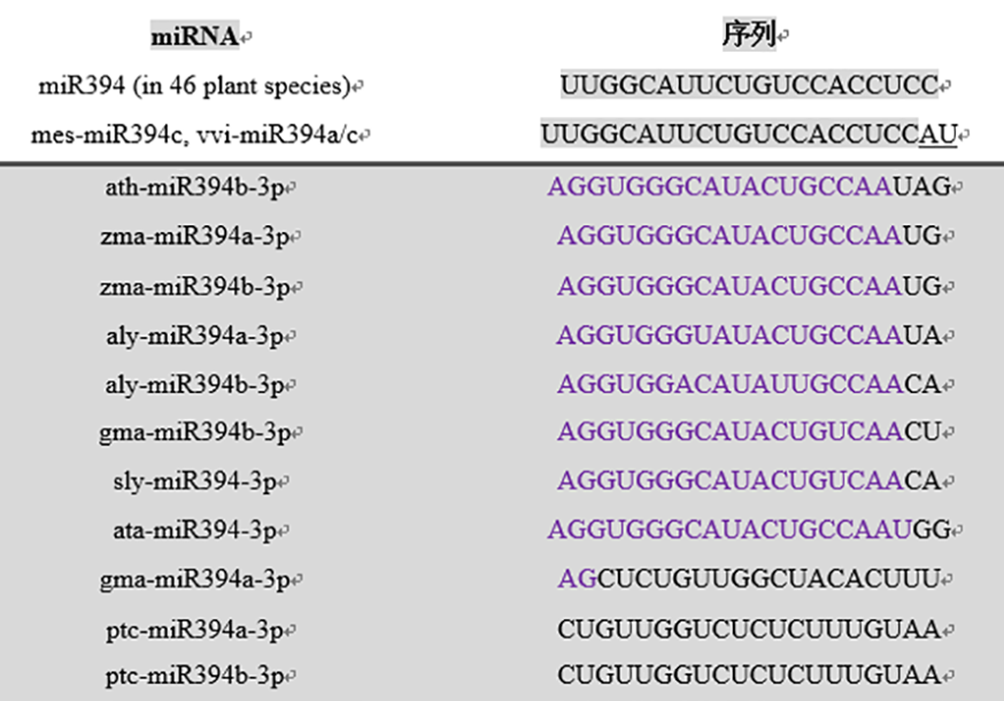

Supplement: FIGURE S1 — Homolog of miR394 in plant species. [file Image_1.TIF]

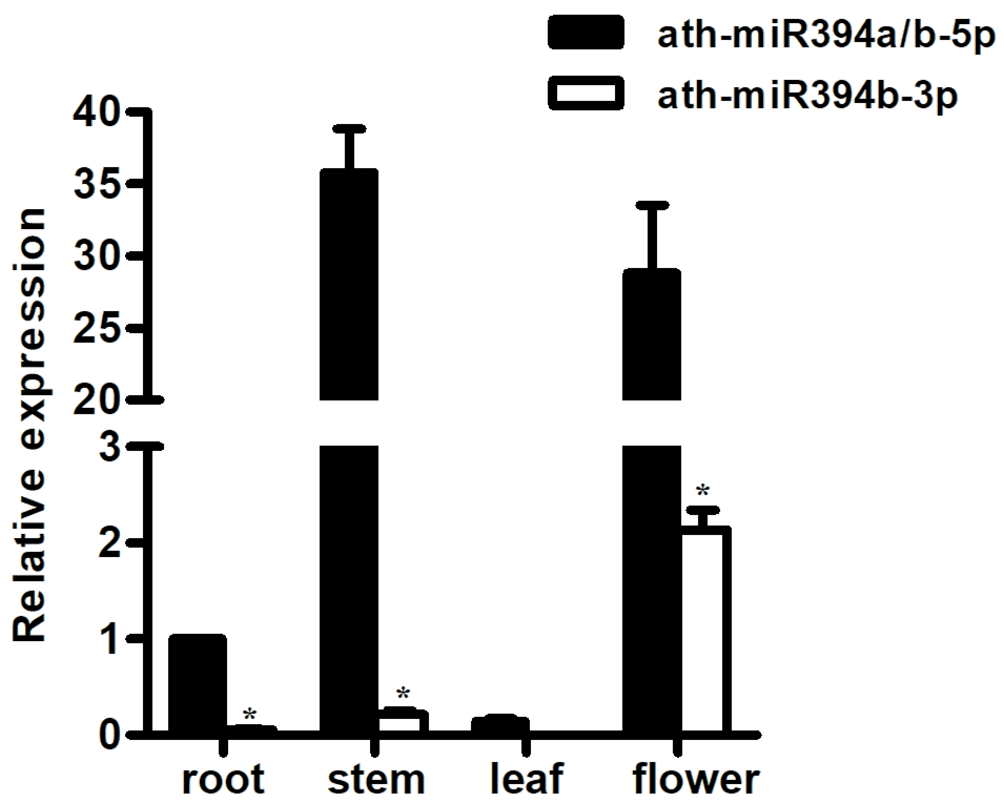

Supplement: FIGURE S2 — Different expression levels in 4 Arabidopsis tissues between miR394-5p and miR394-3p. Asterisks indicate a significant difference (∗P < 0.05). [file Image_2.TIF]

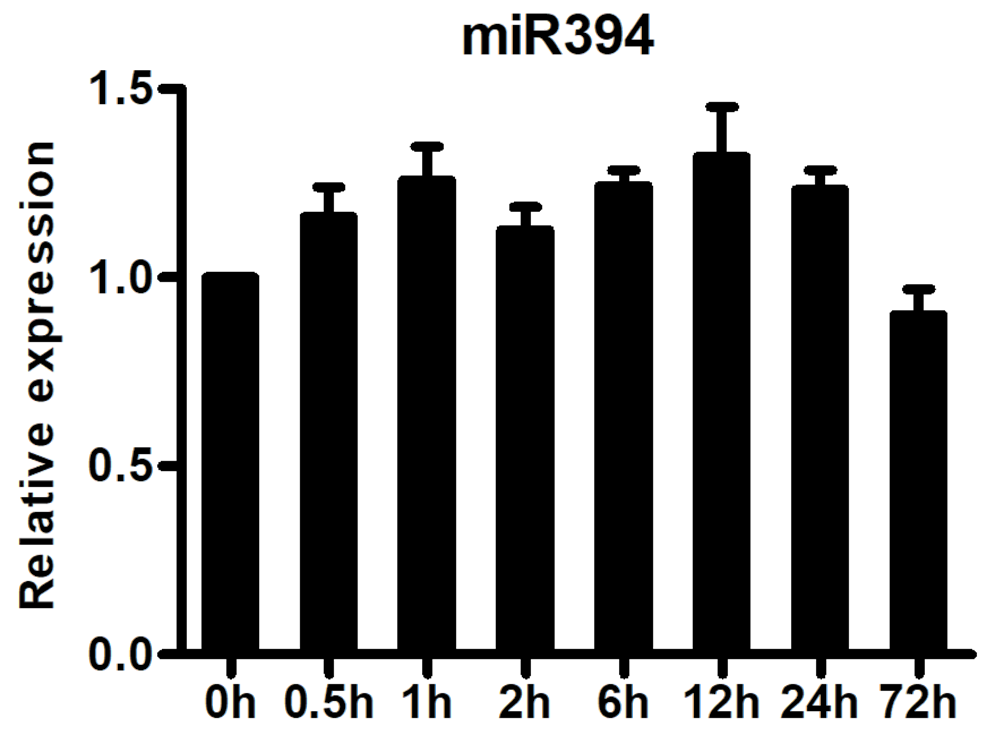

Supplement: FIGURE S3 — Expression pattern of miR394 in mock-infected tomato leaves. [file Image_3.TIF]
